# Results of office-based fluid-air exchange for postvitrectomy hemorrhage in diabetic retinopathy

**DOI:** 10.5935/0004-2749.2022-0334

**Published:** 2024-02-23

**Authors:** Gee-Hyun Kim, Mee Yon Lee

**Affiliations:** 1 Department of Ophthalmology, Seoul St. Mary’s Hospital, College of Medicine, The Catholic University of Korea, Seoul, Republic of Korea; 2 Department of Ophthalmology, Uijeongbu St. Mary’s Hospital, College of Medicine, The Catholic University of Korea, Gyeonggi-do, Republic of Korea

**Keywords:** Diabetic retinopathy, Vitrectomy, Vitreous body, Vitreous hemorrhage, Hemostatic techniques

## Abstract

**Purpose:**

This study aimed to evaluate the efficacy and clinical outcomes of a one-way fluid-air exchange procedure for the treatment of postvitrectomy diabetic vitreous hemorrhage in patients with proliferative diabetic retinopathy.

**Methods:**

This retrospective study included 233 patients with proliferative diabetic retinopathy, who underwent vitrectomy. A one-way fluid-air exchange procedure was performed in 24 eyes of 24 (10.30%) patients with persistent vitreous cavity rebleeding after the operation. Preprocedural and postprocedural best-corrected visual acuity values were achieved. Complications occurring during and after the procedure were analyzed.

**Results:**

Significant visual improvement was observed 1 month after the one-way fluid-air exchange procedure (2.62 ± 0.60 LogMAR at baseline vs. 0.85 ± 0.94 LogMAR at postprocedure, p<0.0001). Moreover, 19 (79.17%) eyes needed the procedure once, and 5 (20.83%) eyed had the procedure more than twice. In 3 (12.50%) eyes, reoperation was eventually required because of persistent rebleeding despite several fluid-air exchanges. No complication was observed during the follow-up.

**Conclusions:**

The one-way fluid-air exchange procedure can be an excellent alternative to re-vitrectomy for patients with proliferative diabetic retinopathy suffering from postvitrectomy diabetic vitreous hemorrhage by removing the hemorrhagic contents directly and achieving fast recovery of visual function without apparent complications.

## INTRODUCTION

Proliferative diabetic retinopathy (PDR) may need sur-gical intervention with vitrectomy to improve visual acuity and reduce the risk of vision loss in cases of nonclearing vitreous hemorrhage (VH), tractional retinal detachment (TRD) threatening the macula, or combined with rhegmatogenous RD (RRD)^(1-3)^. With improvements in vitrectomy techniques and expanding indications, the overall rate of vitrectomies is increasing. Even though pars plana vitrectomy (PPV) has become an effective treatment option for complicated PDR, persistent or recurrent vitreous cavity hemorrhage remains a common problem after vitrectomy for PDR. Some recurrent VHs can clear up spontaneously several weeks from their onset, whereas others require further treatment to help absorb the hemorrhage^(4,5)^. Many options are available for surgical intervention, ranging from intravitreal antivascular endothelial growth factor (VEGF) injection to re-vitrectomy. Vitreous cavity hemorrhage can be also managed by an in-office fluid-air exchange (FAE) procedure^(6-11)^.

In this study, we reviewed the utilization of the one-way FAE technique performed for >4 years at our hospital retrospectively to report outcomes of the procedure for managing postvitrectomy diabetic VH (PDVH).

## METHODS

### Search strategy and study selection

We retrospectively reviewed patients who had under-gone 25-gage trans-PPV with air or perfluoropropane (C3F8) gas tamponade because of PDR complications between January 1, 2017, and March 31, 2021. Patients who experienced vitreous cavity rebleeding right after (persistent rebleeding) or sometime after (recurrent rebleeding) the operation without significant absorption, followed by an in-office FAE procedure, were selected. We considered “no significant absorption” when the optic disc remained invisible for >3 weeks because of hemorrhage in the vitreous cavity and chose to conduct an in-office FAE procedure. The study only included the first eye of each patient. Data on baseline demographics, best-corrected visual acuity before and after the procedure, and procedural complications were collected. Before the FAE procedure, an ultrasound scan was performed to determine the severity of VH or determine the presence of retinal breaks. Eyes with RD and persistent or new tractional membranes were excluded. A single-needle vitreous cavity FAE procedure was conducted by the same surgeon (LMY) in an in-office treatment room. Before the procedure, written consent was obtained from all 24 patients.

### Surgical techniques

**Figure 1:**
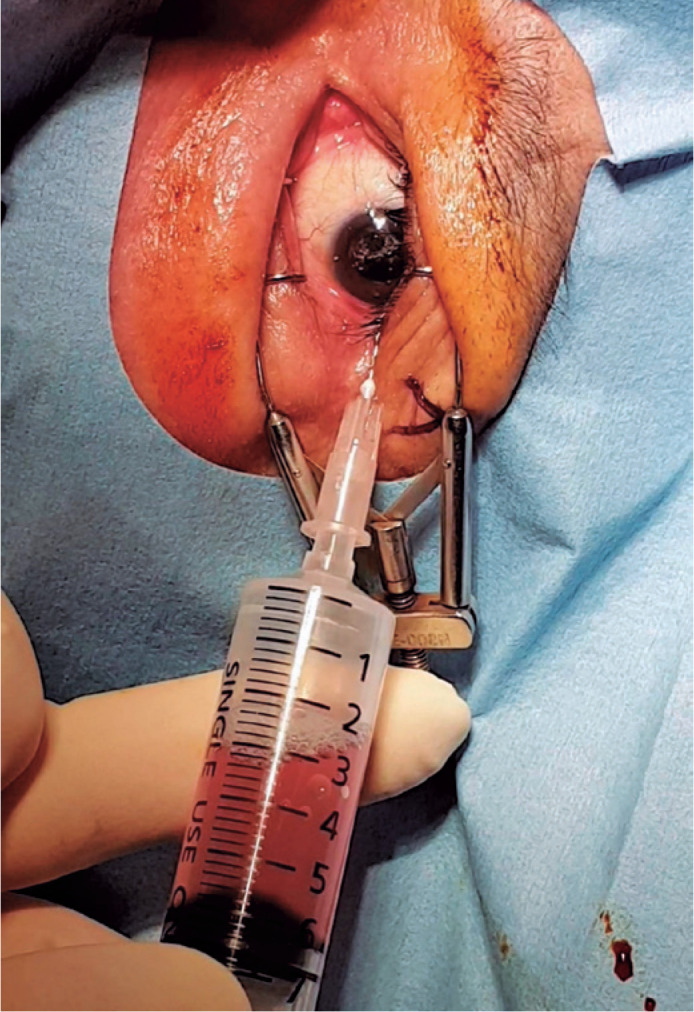
With direct visualization of the needle tip in the vitreous cavity, fluid-air exchange procedure is performed repeatedly at 0.5 mL at a time.

**Figure 2:**
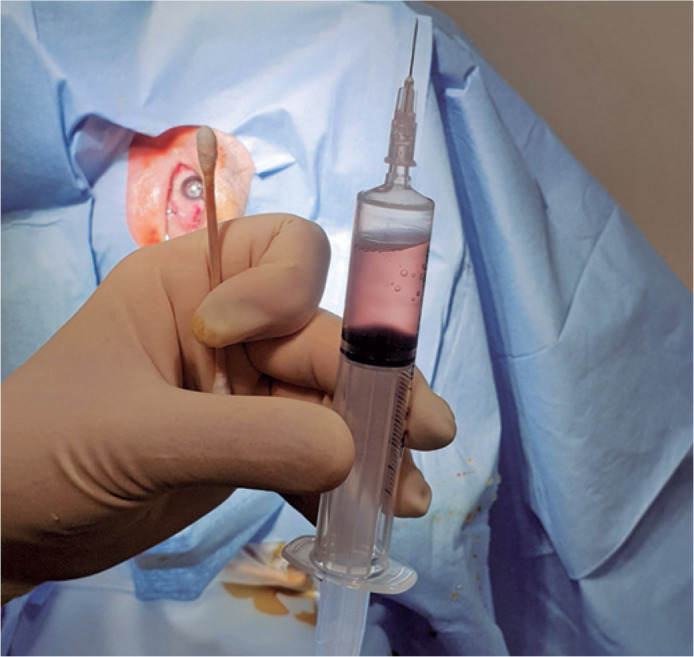
Total 4-5 mL of bloody vitreous fluid is successfully exchanged into the syringe.

Topical anesthesia was initiated. To make the diseased eye dependent, the patient was placed in the lateral decubitus position. The eye was opened with a lid speculum and prepped with a 5% povidone-iodine solution. A 10-mL syringe with a filter (pore size of 0.22 µm) was used to contain 8 mL of sterile air (Figure 1). After filter removal, a 26-gage needle was attached to the syringe. The patient was asked to fix his/her gaze straight forward during the procedure. The syringe needle was introduced into the vitreous cavity 3-3.5 mm posterior to the limbus for a pseudophakic/aphakic eye and 3.5-4 mm posterior to the limbus for a phakic eye on the temporal side of the globe. To avoid injecting air into the subretinal or suprachoroidal space, we ensured that the needle tip was well placed in the vitreous cavity by visualization through the pupil before the FAE procedure. Then, 0.5 mL of air was injected into the vitreous cavity first, and the same amount of existing vitreous fluid was extracted. This procedure was repeated until 4-5 mL of the vitreous fluid was transferred into the syringe. Before the needle was removed, 0.5 mL of air was injected into the vitreous cavity to keep the intraocular pressure (IOP) slightly high. Then, the needle was completely withdrawn (Figure 2). After the procedure, the IOP was qualitatively assessed with finger palpation. The patients were prescribed antibiotic eye drops four times a day for 1 week.

### Statistical approach

The Snellen visual acuity was converted to a logarithmic scale for the minimum angle of resolution (LogMAR) for statistical analysis. According to a previous investigation, hand motion visual acuity and counting finger visual acuity were converted to 3.00 and 2.00 LogMAR, respectively^(12)^. Continuous data were expressed as the mean ± standard deviation (SD). The normality of data distribution was evaluated before choosing the statistical analysis methods. To evaluate the paired differences of the visual acuity, Wilcoxon signed-rank test was used. Correlation analysis was performed to determine any dependency among preoperative parameters. IBM SPSS Statistics version 23 (IBM Corp., Armonk, NY, USA) was used for statistical analysis. The significance level was set at p<0.05.

## RESULTS

### Case characteristics

From January 1, 2017, to March 31, 2021, in our hospital, 233 patients (233 eyes) underwent vitrectomy because of PDR complications. The original indication for vitrectomy was VH or VH combined with TRD. In total, 24 eyes of 24 patients (10.30%) suffered froem recurrent or persistent vitreous cavity rebleeding after the operation. Thus, the one-way FAE procedure was performed. Table 1 presents the details of the baseline characteristics of these patients.

**Table 1:** Demographic and baseline characteristics of the patients

Variables (mean ± SD)	Total
Sex	
Male	18
Female	6
Age, years	57.29 ± 10.19
Disease period of DM, years	13.46 ± 9.86
Days of VH post PPV	192 ± 282
Lens status	
Pseudophakic	21
Phakic	3
Times of fluid-air exchange procedure	
=1	19
=2	3
=3	1
=4	1
Eventual re-vitrectomy required	3

DM= diabetes mellitus; PPV= pars plana vitrectomy; VH= vitreous hemorrhage.

### Demographic and characteristics

The average age of the 24 patients was 57.29 ± 10.19 years. Among these patients, 18 were men and 6 were women. There were significantly (p<0.01) more male patients than female patients in the study group. The average disease period of diabetes was 13.46 ± 9.86 years. In this cohort, 4 (16.67%) patients were anticoa-gulant and/or antiplatelet drug users. However, they stopped using those drugs at least a week before the vitrectomy. Of 24 eyes, 21 were pseudophakic, whereas three were phakic.

The average duration of VH post-PPV was 192 ± 282 days. The mean number of one-way FAE procedures per eye was 1.33, and 19 (79.17%) eyes needed the procedure only once. However, 5 (20.83%) eyes had the procedures not less than twice. Three (12.50%) eyes eventually required re-vitrectomy because of persistent rebleeding despite several FAE attempts and administration of anti-VEGF (bevacizumab).

### Prognosis of fluid-air exchange

To determine the quantitative improvement of visual acuity after the procedure, visual acuity values were converted into LogMAR. The visual acuity appeared to be improved over time after the procedure. The average LogMAR visual acuity at baseline was 2.62 ± 0.60. One week after the procedure, the average LogMAR visual acuity improved (p<0.001) to 1.42 ± 0.95. One month after the procedure, the average LogMAR visual acuity improved (p<0.005) even more 0.85 ± 0.94. Significant (p<0.0001) visual improvement was observed when the LogMAR value at baseline was compared with that at 1 month after the procedure. In all patients, the visual acuity at the final follow-up was still significantly better than that at baseline (Table 2). The correlation analysis also showed no dependency among continuous variables; thus, no confounding variable was identified for the visual outcome.

**Table 2:** Visual acuity outcome before and after the fluid-air exchange procedure

Visual acuity	HM or CF	≤20/200	20/200< <20/50	20/50≤ ≤20/20	LogMAR (Avg ± SD)
Preprocedure	23	0	1	0	2.62 ± 0.60
1 week postprocedure	12	3	4	5	1.42 ± 0.95
1 month postprocedure	7	0	5	12	0.85 ± 0.94

CF= counting finger; HM= hand movement.

No difficulties were encountered when performing the procedure. Lens injury or injury related to cataract formation had not occurred owing to careful intraoperative management; thus, no patient required cataract surgery until the last visit. No other severe complications during and after the procedure occurred.

## DISCUSSION

### VH after diabetic vitrectomy

PPV has become one of the most effective treatment options for complicated PDR; however, VH is a common problem in PDR eyes after vitrectomy^(13,14)^. Recurrent vitreous cavity hemorrhage occurs often even after successful surgical treatment for PDR, and it is a major vision-threatening event. According to Brown et al., recurrent VH is the most common cause of re--vi-trectomy in PDR because 14% of the cases progressed into blindness with no light perception^(15)^. Other studies have found that one-third of postoperative recurrent VH cases with PDR ultimately require FAE or re-vitrectomy to restore visual function^(6,15-19)^.

In many cases, identifying the certain cause of rebleeding after vitrectomy for PDR is difficult. One of the important sources of PVHD is neovascularization at the surgical sclerotomy sites, which are noted in multiple histopathologic studies. Other possible causes of recurrent hemorrhage include residual or recurrent neovascularization of the disc/neovascularization elsewhere, postoperative hypotony, deficient pan-retinal photocoagulation, retinal vessel occlusion, and inadvertent trauma during the operation. Ischemic change of the retinal tissue is the most important factor that contributes to the progression of fibrovascular tissue in cases of diabetic retinopathy after a vitrectomy^(20-23)^. In this study, PDVH occurred in 10%, lower than the rate (37.5%) reported by Yeh et al.^(24)^ However, our follow--up duration was shorter than that reported by Yeh et al. (mean = 19.4 months, median = 21.0 months). The correct management for recurrent VH after vitrectomy should be tailored to each patient.

### Treatments of PDVH

In PDVH, vitreoretinal specialists usually think about whether to observe or intervene surgically. The most common practice to manage such rebleeding after vitrectomy includes repeated intravitreal anti-VEGF injection and/or re-vitrectomy. According to the survey of the American Society of Retina Specialists Patterns and Trends in 2018, 68% of retina specialists preferred anti--VEGF injection, whereas 22% preferred re-vitrectomy. Vitreous cavity hemorrhage can be also treated by either a vitreous cavity lavage or an in-office FAE procedure. However, only 3% of retina specialists chose FAE because of the rising accessibility of vitrectomy and anti--VEGF injection^(25)^.

### Advantages and disadvantages of the one-way FAI

The FAE procedure has the following strengths for PDVH treatment. First, the FAE procedure consists of relatively simple processes; thus, surgeons can easily learn and conduct it even without assistance in the office. Especially, the one-way FAE, which was used in our study, is even simpler than the two-way method. Second, surgeons can obtain more information from the lavage fluid during the procedure and make more adequate decisions for future treatment such as repeated FAE or re-vitrectomy. Third, it is the least invasive way of directly clearing up hemorrhagic contents by entering the vitreous cavity only with a small sclerotomy, which can be self-sealed. Specifically, we adopted a 26-gage needle for FAE, which is thinner than the 25-gage needle used in previous studies. Moreover, topical anesthesia is sufficient for the procedure^(26)^.

However, the FAE procedure has some disadvantages. First, the IOP often changes during the procedure because of its mechanism, and IOP fluctuation can be a risk factor for recurrent and persistent VH^(19)^. If IOP fluctuation is a concern during the procedure, reducing the volume to be replaced at a time could be a solution. According to our results, the one-way FAE is effective and safe despite its simplicity. Second, uncomfortable posture during the procedure might be one of the short-comings. Patients should keep their unnatural lateral decubitus position during the procedure. Third, cataract formation may occur in phakic eyes^(17)^.

Regarding the efficacy of the one-way FAE as a treat-ment option for PDVH, the number of eyes requiring repeated FAE procedures and re-vitrectomy should not be neglected. According to Martin and McCuen, the mean number of procedures for their patients was 1.5, in which 40% of the patients required another vitrectomy by the one-way FAE with a 25-gage needle^(27)^. Han et al. reported that the mean number of FAE procedures for each eye was 1.75, with 25% of eyes requiring re-vitrectomy^(17)^. Behrens et al. performed the two-way FAE, and their average number of procedures was 1.2 of each eye receiving FAE procedures, with 19% of the patients requiring another vitrectomy^(28)^. In the present study, we performed 26-gage one-way FAE, and the mean number of procedures per eye was 1.33, with 12.5% of reoperation, which appear competitive especially since we had not encountered major complications.

As population ages, social and economic costs of healthcare are a growing concern. Physicians must be more aware of the costs of their procedures. In office--based FAE, patients are not transferred the operating room. With mindful patient selection, it can be an efficacious and economical treatment choice in the case of recurrent vitreous cavity hemorrhage after vitrectomy^(28)^.

### Limitations

This study has some limitations. First, this study used a retrospective study design. Second, the sample size was enough to yield statistical significance, but it is difficult to say that it is large. Third, the total follow-up time of the patients was relatively short because patients with more severe VH might already have been treated with a secondary vitrectomy.

Despite the above limitations, our results demonstrate that the one-way FAE procedure could be a preferable option to intravitreal anti-VEGF injection or re--vitrectomy in vitreous cavity rebleeding postvitrectomy in patients with PDR.

Even during the short follow-up time, we could observe fast and significant recovery of visual function after FAE (Table 2). Thus, the visual acuity would improve better after more than a month. Undoubtedly, intravitreal anti-VEGF injection is very convenient. However, retina specialists empirically know that one trial of the injection is usually not enough to clear up the diabetic VH. Moreoer, the interval of injection must be less than a month. However, FAE does not have such a restriction and directly removes hemorrhagic contents. Early visual improvement can be achieved by prompt trials of FAE. Therefore, intravitreal anti-VEGF injection is logically hard to be effective as FAE. As we mentioned, the mean number of FAE per eye was 1.33 in this study. If we suppose “r” as the reoperation rate, then the mean number can be expressed as the sum to infinity of the series mathematically.


$$\begin{array}{rl} \text{ Mean number } & =(1-r)+2r(1-r)+3r^{2}(1-r)+4r^{3}(1-r)+\cdots \\ & =(1-r)\left(1+2r+3r^{2}+4r^{3}+5r^{4}+6r^{5}+7r^{6}+\cdots \right)\\ & =(1-r)\sum_{n=1}^{\infty }nr^{n-1}=(1-r)\frac{1}{(1-r{)}^{2}}=\frac{1}{1-r} \end{array}$$

According to this equation, the re-FAE rate can be calculated as 24.8%. According to Lahey^(29)^ and Suzuki^(30)^, the reoperation rate of vitrectomy ranges from 11% to 13.5%. From a probabilistic point of view, three times FAE equals two times re-vitrectomy in PDVH. Considering cost-effectiveness and safety, FAE is worth trying first instead of another vitrectomy in PDVH.

One-way FAE procedure can effectively remove hemorrhage in the vitreous cavity and improve visual function without causing definite complications in patients with PDR who develop persistent or recurrent hemorrhage after a vitrectomy.
